# Neuroinflammation-associated miR-106a-5p serves as a biomarker for the diagnosis and prognosis of acute cerebral infarction

**DOI:** 10.1186/s12883-023-03241-3

**Published:** 2023-06-27

**Authors:** Wei Du, Lingyan Fan, Juan Du

**Affiliations:** 1 Department of Neurology, Qing Dao Fu Wai Cardiovascular Hospital, Qingdao, 266000 Shandong China; 2 Department of Neurology, Weifang People’s Hospital, Weifang, 261000 Shandong China; 3 Department of Neurology, Yantaishan Hospital, No. 91 Jiefang Road, Yantai, 264001 Shandong China

**Keywords:** MiR-106a-5p, Acute cerebral infarction, Diagnosis, Prognosis, Proinflammatory cytokines

## Abstract

**Background:**

Acute cerebral infarction (ACI) is a common cerebrovascular disease. Previous studies have shown that some abnormally expressed microRNAs (miRNAs) play important roles in ACI. This study aimed to investigate the role of miR-106a-5p in the diagnosis and prognosis of ACI patients, and analyze the regulatory potential of miR-106a-5p on the inflammation of BV-2 microglial cells.

**Method:**

Serum and cerebrospinal fluid (CSF) samples were collected from 98 ACI patients, and the expression of serum miR-106a-5p was analyzed using qRT-PCR. A receiver operating characteristic (ROC) curve analysis was used to evaluate the diagnostic value of miR-106a-5p. The association of miR-106a-5p with ACI prognosis was evaluated using the logistic analysis. In vitro experiments were performed in BV-2 cells by oxygen glucose deprivation (OGD) treatment, and the effects of miR-106a-5p on BV-2 inflammation were assessed using an enzyme linked immunosorbent assay (ELISA).

**Result:**

It was observed that miR-106a-5p was significantly upregulated in the serum and CSF of ACI patients (all *P* < 0.001), and had considerable diagnostic accuracy. The highest serum miR-106a-5p was observed in severe ACI cases, and miR-106a-5p expression was significantly increased in unfavorable prognosis patients. Serum and CSF expression of miR-106a-5p was positively correlated with proinflammatory cytokines in ACI patients, and the inflammation of OGD-induced BV-2 cells was suppressed by miR-106a-5p reduction.

**Conclusion:**

MiR-106a-5p is overexpressed in ACI patients and may serve as a diagnostic and prognostic biomarker for ACI. Furthermore, miR-106a-5p may be involved in ACI progression by regulating neuroinflammation.

## Introduction

Acute cerebral infarct (ACI), also known as acute ischemic cerebral stroke, occurs when blood supply to the brain is suddenly interrupted, resulting in necrosis of brain tissue [1]. ACI patients are mainly caused by atherosclerosis or thrombosis in the arteries that supply blood to the brain, narrowing or even occlusion of the lumen, leading to focal acute cerebral insufficiency [2]. In clinical patients with cerebral infarction is generally divided into mild stage, moderate stage and severe stage, among which initial stage patients are generally conscious, intermediate stage patients appear consciousness obstacle, quadriplegia and other symptoms. Late stages often threaten life and eventually lead to brain death [3]. Studies have shown that the mortality or disability rate of ACI patients in one year is still high, which seriously endangers people’s health [4]. From the perspective of the development process of ACI disease, changes in the cellular biological functions of vascular endothelial cells and vascular smooth muscle cells as well as the enhancement of cellular inflammatory response will promote the occurrence of ACI [5]. After the occurrence of ACI, the microglia in the damaged brain tissue are activated to promote the secretion of inflammatory cytokines, such as interleukin-6 (IL-6), interleukin-1β (IL-1β) and tumor necrosis factor-α (TNF-α), which further aggravates the nerve injury and leads to poor prognosis [6]. This phenomenon provides a new direction for improving the prognosis of ACI patients. Therefore, alleviating neuroinflammation to improve the treatment and prognosis of ACI has still received increasing attention.

At present, the clinical diagnosis of ACI mainly relies on the clinical symptoms of patients and imaging methods, including magnetic resonance imaging (MRI) and computed tomography perfusion imaging (CTPI) [7]. Imaging is important in determining patients having ACI, but CTPI scans are not effective in the early stages of cerebral infarction, and MRI scans cannot detect patients with metal stents [8]. Hence, it is necessary to find novel biomarkers for diagnosis and prognosis of ACI. MiRNAs have become a biomarker attracting much attention in recent decades due to their stable presence in serum [9]. A variety of miRNAs have been proved to be potential biomarkers in a variety of diseases, which are closely related to the occurrence and development of diseases [10]. Studies have shown that miR-106a-5p is an atherosclerotic related miRNA, which not only regulates angiogenesis, but also regulates the activity of vascular endothelial cells and smooth muscle cells [11–13]. Furthermore, it has still been demonstrated that miR-106a-5p is also an inflammation related miRNA [14, 15]. Therefore, we speculated that miR-106a-5p may be involved in the development and progression of ACI and it also had some relationship with microglia-related neuroinflammation.

The purpose of this study was to discuss the expression and clinical value of miR-106a-5p in ACI patients, and to investigate the effects of miR-106a-5p on neuroinflammation in ACI patients and microglia. At the same time, the role of miR-106a-05p in the diagnosis and prognosis of ACI may provide a novel non-invasive biomarker of ACI.

## Materials and methods

### Patient recruitment

A total number of 98 patients with ACI were collected at the Qing Dao Fu Wai Cardiovascular Hospital between 2018 and 2020, including 51 males and 47 females, and 80 healthy volunteers were also enrolled as the control group. The inclusion criteria were as follows: (1) age ≥ 18 years old; (2) patients were diagnosed with ACI by medical history or CTPI; (3) presence of neurological deficits lasting more than 24 h due to ischemic lesions confirmed on conventional MRI of the brain. The exclusion criteria were as follows: (1) pregnant women; (2) patients with transient ischemic attack (TIA); (3) patients with malignant tumors; (4) patients with diseases such as immune infectivity and hematological system alterations. Venous blood was collected from patients after admission and before treatment (less than 4.5 h after disease onset). The serum of patients and volunteers was isolated by centrifuging at 5000 rmp at 4˚C for 20 min, and then collected and stored at ‑80˚C until analysis. Among the 98 ACI patients and 80 healthy volunteers, 29 ACI patients and 12 healthy controls provided cerebrospinal fluid (CSF) samples for further examinations. All healthy controls had no vascular stenosis or atherosclerosis. The patients were divided into severe groups according to the National Institutes of Health Stroke Scale (NIHSS) score, in which NIHSS < 4 was mild group, 4 ≤ NIHSS ≤ 15 was moderate group, and NIHSS > 15 was severe group [16]. This study was approved by the Ethics Committee of Qing Dao Fu Wai Cardiovascular Hospital, and each participant provided written informed consent.

### Cell culture and oxygen glucose deprivation treatment

BV-2 microglial cells were obtained from the Cell Bank of Chinese Academy of Science (Shanghai, China). Cells were cultured in Dulbecco’s modified Eagle’s medium (Hyclone, Shanghai, China; DMEM) supplemented with 10% fetal bovine serum (Gibco; FBS) with penicillin (100 U/ml; Thermo Fisher Scientific, Inc.) and streptomycin (100 µg/mL; Thermo Fisher Scientific, Inc.), and placed in an incubator at 37℃ in a humidified atmosphere containing 5% CO_2_. The culture medium was changed every 24 h, and the cells were subcultured once 48 h.

Oxygen glucose deprivation (OGD) treatment was used to activate BV-2 cells to mimic neuroinflammation conditions in ACI pathogenesis. In brief, BV-2 cells were cultured in serum/glucose-free DMEM medium under a condition of 95% N2 and 5% CO_2_ at 37℃ for 24 h.

### Cell transfection

To regulate the expression of miR-106a-5p in BV-2 cells, miR-106a-5p inhibitor (5’- CUACCUGCACUGUAAGCACUUUU − 3’) and inhibitor NC (5’- CAGUACUUUUGUGUAGUACAA − 3’) were synthesized in Ribobio (Guangzhou, China). According the manufacturers’ protocols, above vectors were transfected into the BV-2 cells with Lipofectamine 2000 (Invitrogen, Carlsbad, CA, USA). Cells were subjected to OGD treatment at 24 h after transfection, thereby downregulating the expression level of miR-106a-5p in activated BV-2 cells.

### Enzyme linked immunosorbent assay

The levels of proinflammatory cytokine in serum samples, CSF and BV-2 cells supernatant were analyzed by Enzyme linked immunosorbent assay(ELISA). The concentrations of inflammatory cytokines IL-6, IL-1β and TNF-α were determined by ELISA kit (Takara, Dalian, China) according to the instructions, respectively.

### RNA extraction and qRT-PCR

Total RNA was isolated from serum samples, CSF samples and BV-2 cells using TRIzol reagent (Invitrogen) and was evaluated by a NanoDrop 2000 (Thermo Fisher Scientific, Waltham, MA, USA) for its concentration and quality, according to manufacturer’s instructions. RT was conducted to synthesize cDNA from 1 µg RNA, using a PrimeScript RT reagent kit (Takara Bio, Inc.). The cDNA was used as template for qPCR, which was carried out to assess the expression levels of miR-106a-5p using SYBR-Green I Master Mix Kit (Invitrogen; Thermo Fisher Scientific, Inc.) and the 7300 Real-Time PCR system (Applied Biosystems; Thermo Fisher Sciences, Inc.). U6 was used as an endogenous control, and the final expression value was calculated using the 2^−ΔΔCt^ method [17].

### Prognosis evaluation methods

Modified Rankin scale (mRS) is used to measure and evaluate the neurological function recovery status of stroke patients [18].The outcomes of the ACI patients were followed up by recording the mRS score at 3 months after disease onset by outpatient and telephone. The mRS score range 0–2 was defined as favorable prognosis, and the range of 3–6 was defined as unfavorable prognosis. According to the mRs score, the patients were divided into favorable prognosis group and unfavorable prognosis group.

### Statistical analysis

The experimental data obtained in this study were presented as mean ± standard deviation (SD) and were analyzed using SPSS version 21.0 (IBM Corp.) and GraphPad Prism version 7.0 software (GraphPad Software, Inc.). The comparisons between groups were performed via Student’s t test, one-way ANOVA with Tukey’s multiple comparison test or Chi-square test. Pearson analysis was used to estimate the correlation between groups. Furthermore, receiver operating characteristic (ROC) curves were used to evaluate the accuracy of miR-106a-5p level in the diagnosis of ACI. Logistics analysis was used to evaluate the prognostic value of miR-106a-5p in ACI. *P* < 0.05 was considered as significant.

## Result

### Baseline characteristics of the participants

In this study, a total of 98 patients with ACI and 80 healthy controls were included. The clinical information of patients and healthy controls is shown in Table 1. There were no significant differences in age, sex, body mass index (BMI), smoking, drinking, hypertension and hyperlipidemia between the two groups (all *P* > 0.05). In addition, the NIHSS of ACI patients was 12.05 ± 5.43.

**Table 1:** Clinical information of the patients and controls

Characteristics	Control group (n = 80)	ACI group (n = 98)	*P* value
Age (years)	62.18 ± 7.64	62.11 ± 6.75	0.954
BMI (kg/m^2^)	23.87 ± 3.58	24.23 ± 3.15	0.474
Male	51	64	0.829
Smoking	30	40	0.652
Drinking	29	42	0.371
Hypertension	44	62	0.264
Hyperlipidemia	31	41	0.676
Diabetes	28	37	0.704
NIHSS	-	12.05 ± 5.43	-

BMI: Body Mass Index; NIHSS: National Institutes of Health Stroke Scale

### Differential expression of miR-106a-5p in patients with ACI

**Fig. 1:**
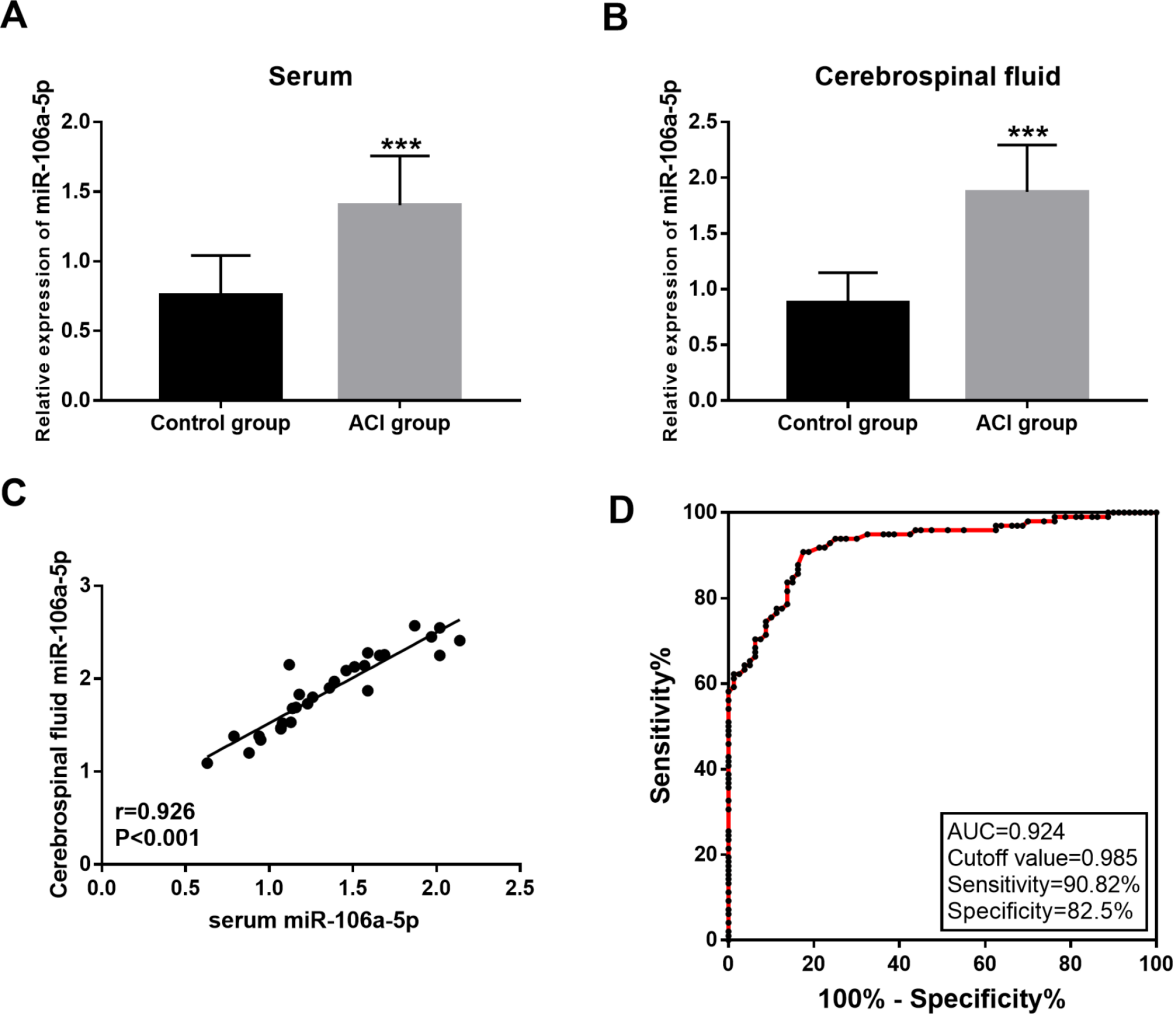
Differential expression of miR-106a-5p in patients with ACI. **A** and **B** Comparison of the serum and CSF levels of miR-106a-5p in patients with ACI and the healthy control. ^***^P < 0.001 **C.** The expression of miR-106a-5p in serum was positively correlated with the expression of miR-106a-5p in CSF. **D.** Results of receiver operating characteristic curve (ROC) to evaluate the sensitivity and specificity of miR-106a-5p for the diagnosis of ACI.

QRT-PCR results showed that compared with the control group, the expression level of miR-106-5p was significantly increased in the serum of ACI patients (*P* < 0.001; Fig. 1A), and the expression level of miR-106-5p was similarly upregulated in the CSF of ACI patients (*P* < 0.001; Fig. 1B). Pearson analysis showed a significant positive correlation between serum miR-106a-5p level and CSF miR-106a-5p level (r = 0.926, *P* < 0.001; Fig. 1C). Furthermore, the ROC curve based on serum miR-106a-5p level showed that the area under curve (AUC) was 0.924, indicating that serum miR-106a-5p had a high diagnostic accuracy in ACI patients (Fig. 1D).

### Relationship between serum miR-106a-5p and ACI severity

**Fig. 2:**
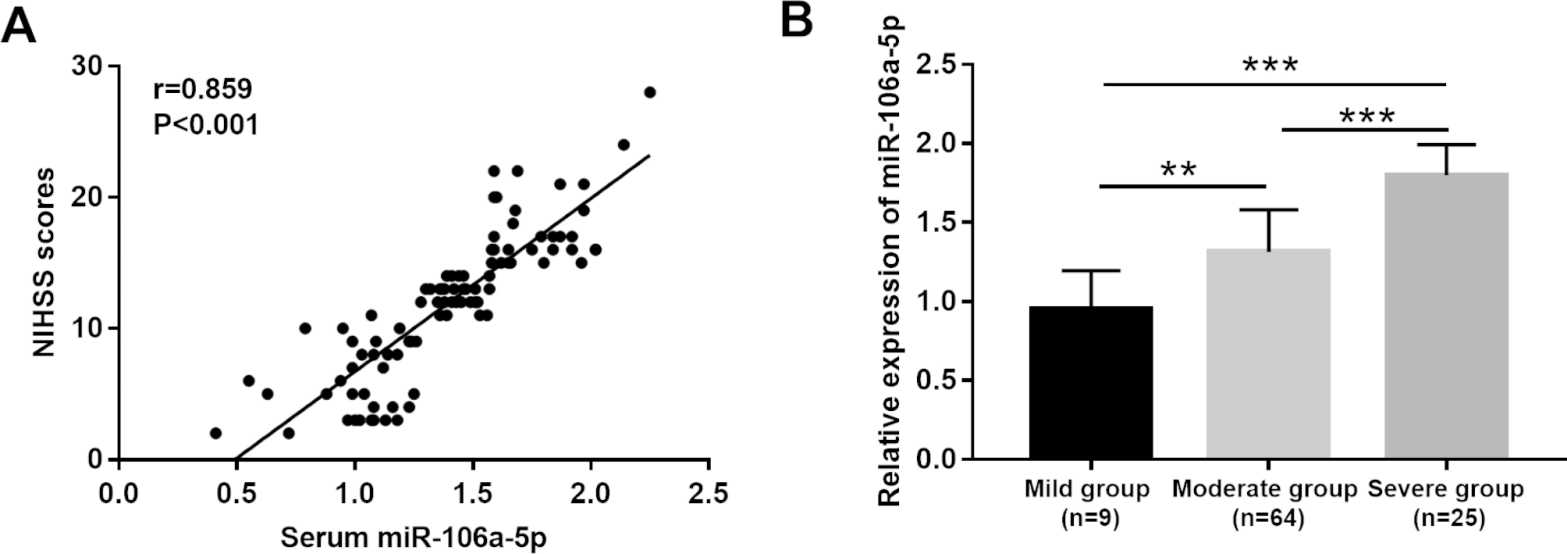
Relationship between serum miR-106a-5p and ACI severity. **(A)** The expression of serum miR-106a-5p was positively correlated with NIHSS score. **(B)** Patients with higher NIHSS scores had higher miR-106a-5p expression levels. ^**^P < 0.01, ^***^P < 0.001

Pearson analysis showed a significant positive correlation between serum miR-106a-5p and NIHSS scores (r = 0.859, *P* < 0.001; Fig. 2A). In this study, the severity of the included patients was grouped according to NIHSS scores. There were 9 cases in mild group, 64 cases in moderate group, and 25 cases in severe group. By comparing the expression levels of miR-106a-5p among the three groups of ACI patients, it was found that the expression level of miR-106a-5p increased with the increase of NIHSS scores, and the patients in the severe group had the highest level of miR-106a-5p (all *P* < 0.01; Fig. 2B).

### Serum high miR-106a-5p levels predict poor prognosis in ACI patients

**Fig. 3:**
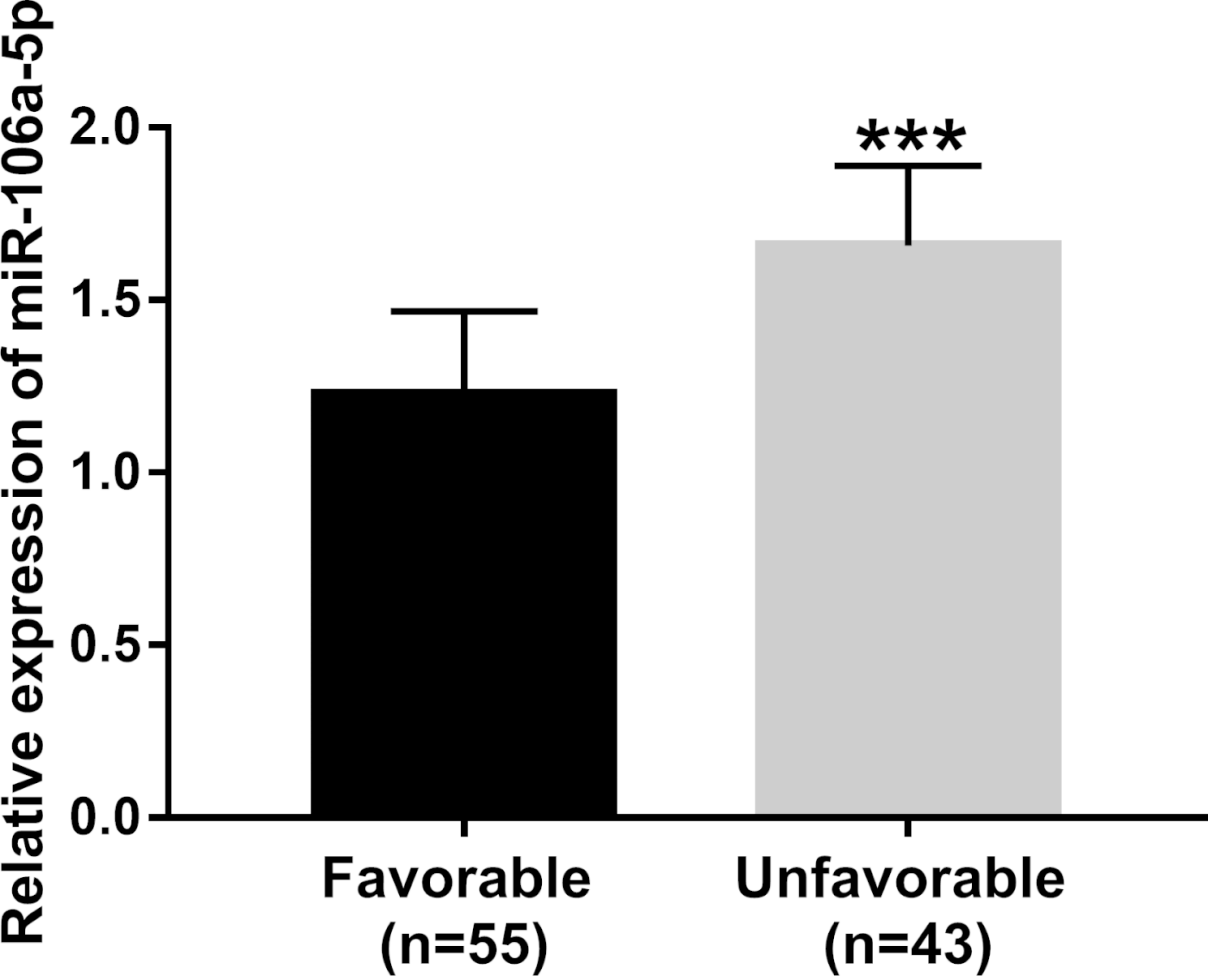
Serum high miR-106a-5p levels predicts poor prognosis in ACI patients. Compared with the favorable group, the expression level of miR-106a-5p was significantly increased in the unfavorable group. ^***^P < 0.001

In this study, the prognosis of patients was evaluated by mRS scores, and 55 ACI patients had favorable prognosis, and 43 ACI patients had unfavorable prognosis. We found that miR-106a-5p was overexpressed in the unfavorable prognosis group compared with the good prognosis group (*P* < 0.001, Fig. 3). In addition, univariate logistics analysis revealed that higher miR‑106a-5p expression (odds ratio (OR), 2.218; 95% confidence interval (CI), 1.528–3.014; *P* = 0.006) and larger NIHSS (OR, 2.401; 95% CI, 1.623‑3.228; *P* < 0.001) were significantly associated with poor prognosis in patients with ACI. Moreover, multivariate analysis identified miR‑106a-5p expression (OR, 2.207; 95% CI, 1.510–2.894; *P* = 0.009) and NIHSS (OR, 2.344; 95% CI, 1.618‑3.047; *P* = 0.002) as independent prognostic factors for the prognosis of patients with ACI (Table 2).

**Table 2:** Logistics analysis for miR-106a-5p expression in patients with ACI

Variables	Univariate analysis		Multivariate analysis	
	OR (95% CI)	*P*	OR (95% CI)	*P*
Age	1.316 (0.668–1.994)	0.692	-	-
BMI	1.089 (0.516–1.489)	0.865	-	-
Male	1.132 (0.761–1.555)	0.604	-	-
Smoking	1.218 (0.758–1.738)	0.332		
Drinking	1.229 (0.773–1.866)	0.298	-	-
Hypertension	1.342 (0.752–2.028)	0.236	-	-
Hyperlipidemia	1.451 (0.857–1.988)	0.158	-	-
Diabetes	1.459 (0.811–2.177)	0.237	-	-
NIHSS	2.218 (1.528–3.014)	0.006	2.207 (1.510–2.894)	0.009
miR-106a-5p	2.401 (1.623–3.228)	< 0.001	2.344 (1.618–3.047)	0.002

BMI: Body Mass Index; NIHSS: National Institutes of Health Stroke Scale; OR: odds ratio; CI: confidence interval

### Correlation of miR-106a-5p with proinflammatory cytokines in patients with ACI

The correlation between miR-106a-5p expression level and inflammatory cytokines (IL-1β, IL-6 and TNF-α) was analyzed in serum samples and CSF of patients with ACI. The analysis results showed that serum miR-106a-5p were positively correlated with IL-1β (r = 0.598; *P* < 0.001), IL-6 (r = 0.598; *P* < 0.001) and TNF-α (r = 0.598; *P* < 0.001), respectively. Similarly, the analysis results still showed that CSF miR-106a-5p was positively correlated with IL-1β (r = 0.712; *P* < 0.001), IL-6 (r = 0.697; *P* < 0.001) and TNF-α (r = 0.846; *P* < 0.001) (Table 3).

**Table 3:** Correlation of miR-106a-5p with proinflammatory cytokines in patients with ACI

Cytokines	Serum miR-106a-5p	
	r value	P value
IL-1β	0.598	< 0.001
IL-6	0.513	< 0.001
TNF-α	0.632	< 0.001
Cytokines	Cerebrospinal fluid miR-106a-5p	
	r value	P value
IL-1β	0.712	< 0.001
IL-6	0.697	< 0.001
TNF-α	0.816	< 0.001

IL-6, interleukin-6; IL-β, interleukin-1β; TNF-α, tumor necrosis factor-α

### MiR-106a-5p reduction alleviates neuroinflammation in microglia treated with OGD

**Fig. 4:**
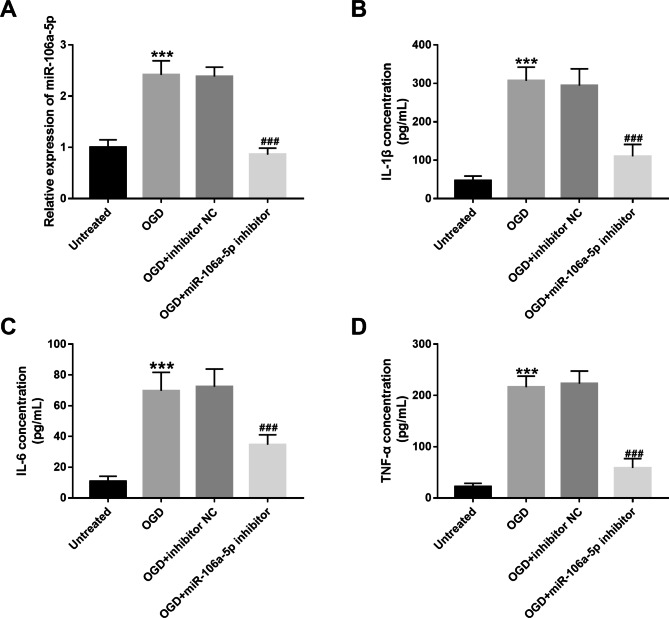
MiR-106a-5p reduction alleviates neuroinflammation in microglia treated with OGD. **A.** MiR-106a-5p inhibitor can reverse the promotion effect of OGD treatment on miR-106a-5p expression in BV-2 cells. **B-D.** After OGD treatment, the levels of inflammatory cytokines were significantly increased, and miR-106a-5p inhibitor could eliminate the promoting effect of OGD treatment on the release of inflammatory cytokines in BV-2 cells. ^***^P < 0.001, ^###^*P* < 0.001; ^*^ compare with untreated, ^#^ compare with OGD.

BV-2 cells were subjected to OGD treatment and miR-106a-5p inhibitor transfection. Our results showed that the expression level of miR-106a-5p was significantly up-regulated in OGD treated BV-2 cells, whereas miR-106a-5p inhibitor can reverse the promotion effect of OGD treated on miR-106a-5p expression in BV-2 cells (all *P* < 0.001, Fig. 4A). Further proinflammatory cytokine level analysis experiments showed that the levels of proinflammatory cytokine (IL-1β, IL-6 and TNF-α) were significantly upregulated in OGD treated BV-2 cells, indicating that cellular inflammation was activated. However, in miR-106a-5p-silenced BV-2 cells, OGD-induced increases in IL-1β, IL-6 and TNF-α levels were all abolished (all *P* < 0.001, Fig. 4B-D).

## Discussion

In this study, we observed abnormal expression of miR-106a-5p in the serum and CSF of ACI patients, which was associated with disease severity. MiR-106a-5p may serve as biomarkers for early diagnosis and clinical prognosis in patients with ACI. In addition, both serum and CSF miR-106a-5p expression were correlated with the inflammation of ACI, and miR-106a-5p inhibition in BV-2 cells led to decreased neuroinflammation.

In recent decades, increasing evidence has shown that circulating microRNAs can be diagnostic markers of ACI [10]. For example, Wu et al. showed that plasma miR-99b levels were down-regulated in ACI patients and that plasma miR-99b levels may be diagnostic markers [19]. Wang et al. reported that miR-497 expression in ACI was positively correlated with inflammatory cytokines, and the level of miR-497 could be used as a biomarker for diagnosis of ACI patients [20]. Zhou et al. showed that miRNA-21 and miRNA-24 in plasma may serve as potential markers for early acute cerebral infarction [21]. The role of miR-106a-5p in central nervous diseases has been discussed in previous studies [14, 22]. Nevertheless, it remains to be examined whether the expression level of miR-106a-5p changes and its role in ACI patients. In this study, the experimental results showed that the expression level of miR-106a-5p was significantly up-regulated in the serum and CSF of ACI patients. And, the expression level of miR-106a-5p in blood was positively correlated with that in CSF. In addition, ROC analysis showed an AUC of 0.924, sensitivity of 90.82% and specificity of 82.5%, indicating that serum miR-106a-5p level is a biomarker with high accuracy in the diagnosis of ACI.

Previous clinical studies have shown that ACI can lead to serious sequelae and most ACI patients have a poor prognosis [23]. Therefore, it is important to search for new and effective prognostic markers. Previous studies have still reported that miR-106a-5p is associated with the prognosis of several diseases, such as head and neck squamous cell carcinoma [24], hepatocellular carcinoma [25] and colon cancer [26]. In the present study, we found that the expression level of miR-106-5p in serum was positively correlated with NIHSS score, and the ACI patients who were in the severe group had the highest expression level of miR-106-5p. NIHSS score is associated with functional outcome as well as mortality in patients with ACI [27]. This indicates that the overexpression of miR-106a-5p is closely related to the poor prognosis of ACI. Meanwhile, the results of logistics analysis showed that the expression level of miR-106-5p and the NIHSS score as independent prognostic factors for ACI. In addition, the patients with ACI were further divided into favorable group and unfavorable group according to the mRs score, and the experimental results showed that the expression level of miR-106a-5p in the unfavorable group was significantly increased. Taken together, these results suggested that miR-106a-5p is a potential novel prognosis biomarker for ACI.

This study further investigated the mechanism of miR-106-5p in ACI patients. Previous studies have shown that miR-106-5p is an inflammation-related miRNA [15]. For instance, Xiang et al. found that miR-106a-5p mimics can reduce IL-1β-induced apoptosis in osteoarthritis, and the combination therapy of baicalein (BAI) + miR-106a-5p has a stronger anti-inflammatory effect than monotherapy [15]. MiR-106a-5p can regulate interleukin 17 (IL-17)-producing T helper cell differentiation in Multiple sclerosis (MS) patients [14]. At the same time, we found that the level of inflammatory cytokines was closely related to the progression of ACI [6]. Ischemic hypoxia changes after cerebral infarction (CI), in which many proinflammatory cytokine such as IL-1, IL-6 as well as cytokines such as TNF-α are significantly released in the body before the inflammatory cell cascade occurs [28]. And the overexpression of proinflammatory cytokine can lead to a cytotoxic response, promoting the formation and increase of infarct lesions [29]. Therefore, we speculated that miR-106a-5p is involved in the progression of ACI by regulating the level of inflammatory cytokines. In the present study, OGD treatment and miR-106a-5p inhibitor transfect microglial cells were used for the experiments. Our results showed that the expression levels of proinflammatory cytokine were positively correlated with the expression levels of miR-106a-5p in serum and CSF. And, in cells activated to inflammatory response by OGD treatment, knockdown of miR-106a-5p expression level could attenuate the proinflammatory cytokine release level of OGD treated cells. Therefore, miR-106a-5p may affect the disease progression of ACI by regulating the release levels of proinflammatory cytokine, which indicates a new ideaof the treatment and prognosis of ACI.

Although this study provided a novel insight into the clinical and functional role of miR-106a-5p in ACI, there are some limitations, such as small sample size, especially the limited CSF samples collected, and lack of animal experiments. We hope to collect sufficient samples and conduct animal experiments in future studies to further confirm our conclusion. In addition, this study used mouse-derived microglia but not human-derived cells to perform the in vitro studies, which may decrease the potential to find more convincing and interesting results. Thus, future studies are necessary to deeply investigate the mechanisms in human-derived microglia.

In conclusion, our results suggest that serum and CSF miR-106a-5p is up-regulated in patients with ACI, and that miR-106a-5p can be used as novel diagnostic and prognostic biomarker for ACI. At the same time, both serum and CSF miR-106a-5p levels are correlated proinflammatory cytokines in ACI patients, and miR-106a-5p reduction suppresses BV-2 cell inflammation, which suggested that miR-106a-5p may be involved in ACI progression by regulating neuroinflammation. Our data may provide evidence for a new biomarker and therapeutic target of ACI.

## Data Availability

The datasets generated and analysed during the current study are not publicly available due [CONFIDENTIALITY AGREEMENT] but are available from the corresponding author on reasonable request.

## Abbreviations

ACI: Acute cerebral infarction
miRNAs: microRNAs
CSF: Serum and cerebrospinal fluid
ROC: receiver operating characteristic
OGD: oxygen glucose deprivation
ELISA: immunosorbent assay
IL-6: interleukin-6
IL-1β: interleukin-1β
CTPI: computed tomography perfusion imaging
TIA: transient ischemic attack
CSF: cerebrospinal fluid
NIHSS: Health Stroke Scale
mRS: Modified Rankin scale
CI: cerebral infarction
